# Rituximab as Single Agent in Primary MALT Lymphoma of the Ocular Adnexa

**DOI:** 10.1155/2015/895105

**Published:** 2015-09-06

**Authors:** Ombretta Annibali, Francesca Chiodi, Chiara Sarlo, Magdalena Cortes, Francesco M. Quaranta-Leoni, Carlo Quattrocchi, Antonella Bianchi, Stefano Bonini, Giuseppe Avvisati

**Affiliations:** ^1^Department of Haematology, University Campus Bio-Medico, Via A. del Portillo 200, 00128 Rome, Italy; ^2^Department of Ophthalmology, University Campus Bio-Medico, Via A. del Portillo 200, 00128 Rome, Italy; ^3^Orbital and Adnexal Service, Villa Tiberia Hospital, Via Emilio Praga 26, 00137 Rome, Italy; ^4^Department of Radiology, University Campus Bio-Medico, Via A. del Portillo 200, 00128 Rome, Italy; ^5^Department of Anatomopathology, University Campus Bio-Medico, Via A. del Portillo 200, 00128 Rome, Italy

## Abstract

Ocular Adnexal Lymphomas are the first cause of primary ocular malignancies, and among them the most common are MALT Ocular Adnexal Lymphomas. Recently systemic immunotherapy with anti-CD20 monoclonal antibody has been investigated as first-line treatment; however, the optimal management for MALT Ocular Adnexal Lymphomas is still unknown. The present study evaluated retrospectively the outcome of seven consecutive patients with primary MALT Ocular Adnexal Lymphomas, of whom six were treated with single agent Rituximab. All patients received 6 cycles of Rituximab 375 mg/mq every 3 weeks intravenously. The overall response rate was 100%; four patients (67%) achieved a Complete Remission, and two (33%) achieved a partial response. In four patients an additional Rituximab maintenance every 2-3 months was given for two years. After a median follow-up of 29 months (range 8–34), no recurrences were observed, without of therapy- or disease-related severe adverse events. None of the patients needed additional radiotherapy or other treatments. Rituximab as a single agent is highly effective and tolerable in first-line treatment of primary MALT Ocular adnexal Lymphomas. Furthermore, durable responses are achievable with the same-agent maintenance. Rituximab can be considered the agent of choice in the management of an indolent disease in whom the “quality of life” matter is of primary importance.

## 1. Introduction

Ocular Adnexal Lymphomas (OALs) are a heterogeneous group of lymphoproliferative neoplasms involving the orbital anatomic region and its structures: lacrimal glands, extraocular muscles, conjunctiva, eyelids, and the orbit itself. They are the main cause of primary ocular malignancies, accounting for more than 50% of cases [1], and represent about 1-2% of Non Hodgkin Lymphomas (NHL) and 8% of Extranodal NHLs. Extranodal Marginal Zone Lymphoma (MALT lymphoma) is the most common histology of primary OALs (50–80% of cases), followed by Follicular Lymphoma (10–20%), Diffuse Large B-cell Lymphoma (8%), and other less common low grade B-cell NHL, with rare incidence of aggressive, T-cell, and Hodgkin lymphomas. The great majority (92%) of Extranodal Marginal Zone OALs are primarily ocular, while other histologies, in particular high grade diseases, in many cases involve ocular structures primarily or secondarily [2]. Recent data about OALs show that incidence has been increasing over the last decades [3, 4]. The postulated origin of these neoplasms is the postgerminal-center memory B cell, which has the capacity to differentiate into marginal zone cells and plasma cells.

Treatment, for lymphoproliferative disorders involving ocular adnexa, may be widely different. In fact, while high grade or multicentric forms of lymphomas invariably need systemic polychemotherapy, indolent and localized lymphomas like MALT OALs, which represent the vast majority of the cases, may not need an intensive systemic treatment. In the past decades many treatments for MALT OALs were used: surgical resection, antibiotic therapy, cryotherapy, radiotherapy, and interferon alpha. More recently immunotherapy with Rituximab emerged as an interesting option, because of its safe toxicity profile and good tolerability together with the chance of durable remissions. However, the real value of Rituximab immunotherapy in primary MALT OALs is not well established yet. For this reason, we evaluated the efficacy of systemic Rituximab immunotherapy in 7 consecutive patients with primary MALT OAL.

## 2. Patients and Methods

**Figure 1:**
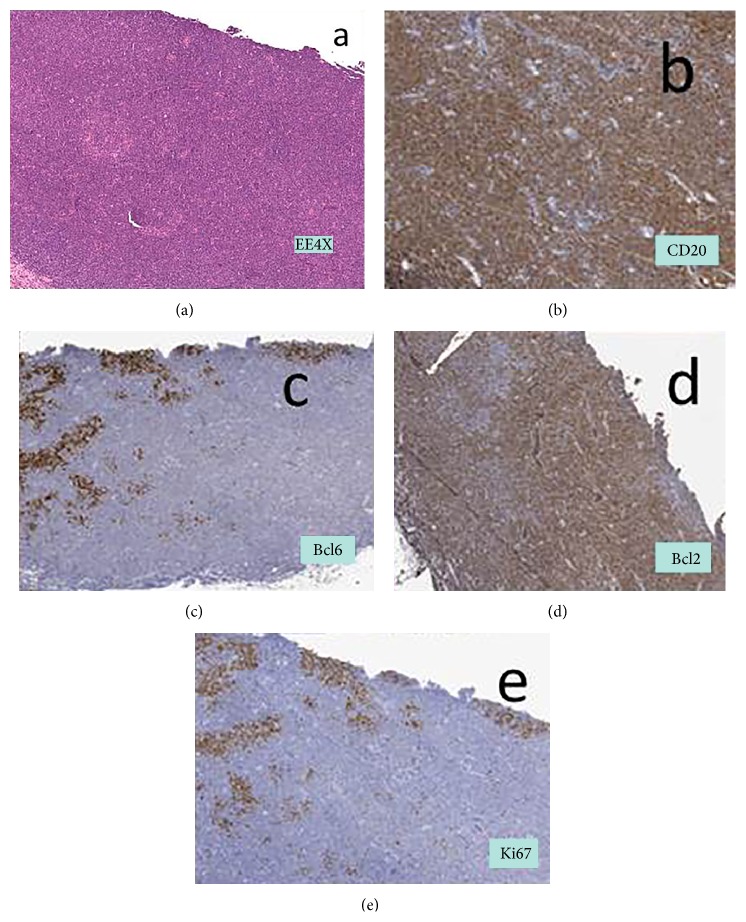
Characteristic diffuse infiltrate of lymphoid element with small nuclei (a) positive to immunohistochemical staining for CD20 (b) and bcl2 (d) and negative for bcl6 (c) with low ki67 (e). The infiltrate surrounds reactive follicles evidenced by positivity for bcl6 and negativity for bcl2 associated with high ki67.

From 2004 to 2014 we observed 11 consecutive OALs. Of these, 7/11 (63% of cases) were MALT lymphomas, 2/11 (18%) were Mantle Cell Lymphomas, 1/11 (9%) was a Follicular Lymphoma, 1/11 (9%) was a Marginal Zone B-cell lymphoma. We included in this analysis 7 consecutive patients with primary histologically diagnosed CD20+ MALT OALs according to the WHO 2008 classification [5], Ann Arbor staging system IE, treated with Rituximab immunotherapy alone between March 2012 and December 2014. One of these patients, showing an increased uptake in PET scans, was excluded from the study because of a relatively aggressive bilateral disease and underwent treatment with R-COMP polychemotherapy. None of the patients enrolled was previously treated. For each of the 6 eligible patients we recorded age, sex, laterality, affected tissue, presenting signs and symptoms, serologic markers, dose and response to Rituximab treatment, follow-up period, complications, and survival status. At the diagnosis in all patients an incisional or excisional biopsy with immunohistochemical staining for histopathologic definition was performed. In Figure 1, we showed characteristic diffuse infiltrate of lymphoid element surrounding reactive follicles. Moreover, a complete ophthalmic examination, a Total Body Computer Tomography (CT) scan, a Positron Emission Tomography (PET) scan, and an Esophagogastroduodenoscopy and Colonscopy were performed to exclude any systemic involvement. To define the tumor extension and its relationship with close structures, a Magnetic Resonance Imaging (MRI) of the orbital region was also performed. Bone marrow biopsy was not performed since previous studies have demonstrated any benefit in the staging of MALT OALs [6]. All patients received six cycles of systemic Rituximab immunotherapy at a dose of 375 mg/mq intravenously, every 3 weeks. Three patients (50%) were evaluated with an interim MRI scan after three cycles. In all patients after the sixth cycle the response to treatment was assessed with an MRI and a PET scan to define three grades of response: complete, partial, or stable disease. Response to treatment was evaluated on the basis of clinical, radiologic, and pathologic criteria, with the definition of a complete response (CR), partial response (PR), stable disease (SD), and progressive disease (PD) referring to the international response criteria for malignant lymphoma [7]. Response was evaluated at the end of treatment program (after 6 courses).

## 3. Results

**Figure 2:**
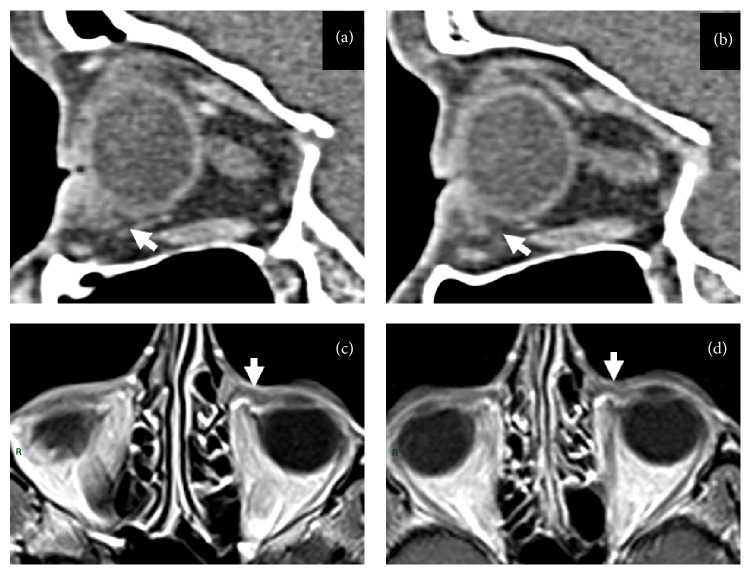
Imaging signs of B cell lymphoma response to therapy. ((a) and (b)) Computed tomography sagittal reformats show focal thickening of the anteroinferior peribulbar conjunctiva on the left side (white arrow in (a)). Compared to the same site in the right orbit (arrow in (b)) where peribulbar hypodense fat tissue is present. ((c) and (d)) Magnetic resonance axial postgadolinium T1-weighted images before (c) and after (d) treatment. Note the size reduction of the focal peribulbar tissue on the left medial conjunctiva (white arrows in (c) and (d)).

In Table 1 patients and disease features, treatment, and outcome are summarized. Median age was 57 years (range 37–67 years), four females, and two males were enrolled, with a female/male ratio of 4/2. Five patients (83%) presented with unilateral disease, and one (17%) with bilateral involvement. In four patients (66%) the disease involved the conjunctiva, and in two patients (33%) it had an orbital localization. In none of the cases there was presence of systemic disease. The most common presenting signs and symptoms were ocular swelling (four patients, 67%), conjunctival erythema (17%), and xerophthalmia (17%). All the patients were diagnosed with biopsy-proven histological examination which resulted in CD20+. The diagnosis in all patients was Mucosa-Associated Lymphoid Tissue OAL. None but one of the patients was previously treated for their ocular disease. The pretreated patient had received interferon *α*-2b, that was rapidly discontinued (after few days) for intolerance. All patients received 6 cycles of Rituximab systemic immunotherapy at the standard dose of 375 mg/mq every 21 days. With the exception of one patient having a Varicella Zoster Virus (VZV) reactivation (Ramsay Hunt syndrome) treated with acyclovir per os, no systemic or ocular relevant side effects were observed. Of the three patients who underwent an interim evaluation by MRI scan. As showed in Figure 2, one had a complete response (CR), the second had a partial response (PR), and in the third case a stable disease (SD) was demonstrated. After the sixth Rituximab cycle, four patients (67%) achieved a CR, and the remaining two patients (33%) achieved a PR. On the whole, all the six patients were responders to Rituximab treatment. After the completion of this treatment, four patients started a maintenance therapy with Rituximab 375 mg/mq every 2-3 months for two years. Of them, three are still in CR and one maintains a PR. Rituximab maintenance was well tolerated in all patients, except one case who presented herpetic keratitis (he was the same patient who had had the VZV reactivation). None of the patients underwent local radiotherapy. After a median follow-up of 29 months (range 8–34), no recurrences of MALT lymphoma were observed, nor treatment or disease-related deaths. Five of the six patients are alive at the time of this analysis (January 2015); one patient died because of lung cancer relapse and could not start Rituximab maintenance; this patient had achieved a PR of its OAL. Maintenance treatment with Rituximab is still ongoing in 4/5 alive patients.

## 4. Discussion

Primary localized MALT OALs are malignancies having indolent behavior, usually associated with a favorable prognosis, rare lymphoma-related deaths, and a non- or oligosymptomatic course. Thus, the treatment strategy should be chosen considering both efficacy profile and toxic effects. Beside conventional treatments like surgery, chemotherapy, and radiotherapy, associated with potential systemic and local damage, other less toxic strategies have been studied, including intralesional injection of Interferon *α*-2b, brachytherapy, and antibiotic therapy. In particular, surgical excision alone as treatment of OALs is followed by local relapse and by disseminated extraocular disease [8–10]; therefore, the role of surgery is currently limited only to diagnostic biopsy.

Radiotherapy plays an intriguing role in the treatment of OALs. It has been proven that radiotherapy is capable of inducing a local control rate of the disease up to 100% of cases regardless of the histologic subtype of the lymphoma and a low recurrence rate ranging between 0% and 15%. Therefore, it is considered to be the standard treatment for low grade OALs localized to the orbit [11–15]. Moreover, in 17%–65% of patients, a lead shield to protect the cornea was used. Depending on the studies median dose of radiotherapy ranged between 24 and 30.6 Gy and the amount of Gy per fraction varied between 1.5 and 2.5 Gy depending on the study [6, 16–23].

Generally single agent chemotherapy such as chlorambucil or purine analogs (fludarabine, cladribine, and pentostatin) or low toxicity combined regimens such as CVP (cyclophosphamide, vincristine, and prednisone) are utilized for the chemotherapeutic treatment of OALs patients who have or not systemic involvement. The adjunct of chemotherapy to radiotherapy did not add any benefit, and the toxicities rates were similar between the two treatment regimens [9, 24]. Considering the proposed role of Chlamidia psyttaci in the pathogenesis of OAL, an original approach in the treatment of these types of Lymphoma has been that of using antibiotic treatment directed against* Chlamydia psittaci*. This type of antibiotic treatment was firstly proposed by Ferreri et al. who showed an objective clinical response in 80% of treated patients with doxycycline [25]. This result was confirmed by Abramson et al. [26]. On the contrary, Grünberger and colleagues [27] did not observe any positive results in their patients. Finally a further study reported that oral doxycycline led to a positive clinical response in 64% of* Chlamydia psittaci* DNA-positive and 38% DNA-negative OALs [28] leading to the conclusion that results obtained in OALs with doxycycline are variable.

In a recent review on the use of antibiotic therapy in nongastrointestinal MALT lymphoma [29] the cumulative results obtained with the use of doxycycline 100 mg BID for 21 days in a total of 8 studies [25–28, 30–33] were reported. Only 3 of these studies were prospective [25, 28, 30], and one was a case report [33]. Overall, in the prospective studies 70 newly diagnosed OALs were accrued, while the retrospective studies have accrued 58 patients. A further study reported in this review was a prospective study utilizing in 11 OALs patients Clarithromycin 500 mg BID for 6 months [34]. Overall, in these 9 studies 23 patients (18%) achieved Complete Remission, 36 (27%) had partial remission, 55 (42%) had stable disease, and 8 (6%) had a progressive disease accounting for an overall response rate of 45%. Very recently, a complete response was obtained by the use of clarithomycin 500 mg twice per day for 4 weeks in a OALs who refused conventional oncologic therapy and tested negative for all potential bacterial causes of MALT lymphoma proposed so far [35].

In the last years the efficacy of systemic single agent Rituximab immunotherapy has been emphasized in the management of primary MALT OALs, as second-line [36] or first-line [37] treatment. However, because of the rarity of the disease, the available data are not uniform. Larger case series attempt to define treatment outcomes with different agents [23, 38]. Meanwhile the studies available about the use of upfront Rituximab as a single agent are very few [39, 40]. Furthermore, no data were available about the possible use of Rituximab maintenance during the follow-up of MALT OALs. Recently Ardeshna et al. [41] have demonstrated an improved Progression Free Survival (PFS) in indolent lymphomas receiving a 2-year maintenance treatment with Rituximab versus no treatment.

Taking into account the small number of patients enrolled, the first aim of our report is to strengthen the excellent response rate of untreated primary MALT OALs to single agent Rituximab demonstrated in literature. Overall response rate (ORR) was, in fact, 100%, and the quality of response was high for the majority of the cases, reaching a CR in four patients (67%) and a PR in two patients (33%), without recurrence. In our study, differently from other reports, all patients were treated with the same induction schedule (6 cycles of Rituximab 375 mg/mq every 3 weeks).

The second aim is to explore the usefulness of Rituximab maintenance in this specific clinical setting, not investigated yet in any report. In our case series four of the six patients were, after Rituximab induction, subsequently maintained with Rituximab every 2 or 3 months. After a median follow-up period of 29 months from the start of therapy and of 21 months from the start of maintenance treatment, we observed no serious adverse events and all patients maintained the achieved response. A limit of our study is the relatively short follow-up together with the limited enrollment; however, it is the first description of clinical outcome in localized primary MALT OALs patients treated with first-line single agent Rituximab followed by same-agent maintenance.

At the present, the main question regarding the optimal management for localized primary MALT OALs cannot be answered yet, since no prospective randomized trials comparing different upfront treatments have been conducted.

As a local approach, surgical excision can be a weapon to treat encapsulated tumors. However, the risk of an incomplete resection is too high and generally not acceptable according to most authors [42, 43].

Involved-field radiotherapy (IFRT) is the current standard of care and has been widely studied in MALT OALs. In the literature good response patterns (OR 85–100%) and durable local control are reported, though accompanied by ocular short- and long-term adverse effects (conjunctivitis, cataract, xerophthalmia, retinopathy, corneal damage, and vision loss) [16, 21, 44]. As reported also in the study by Sasai et al. [9], IFRT seems associated with a considerable risk of systemic recurrence, while a minor risk is seen with Rituximab treatment. The risk of systemic relapse is higher in bilateral ocular presentation of MALT OALs [45], and this seems to suggest a questionable usefulness of IFRT in bilateral disease. Moreover, there is no universally accepted radiation schedule for patients with OAL, and controversy still exists regarding the optimal radiation dose and fractionation (for most authors, comprised between 20 Gy and 30 Gy). Furthermore, retreatment of the same tissue should be avoided, and the “quality of life” matter, in such an indolent disease, should be considered when efficacy is guaranteed by the less toxic treatments.

Also a watchful waiting approach has been studied in patients with asymptomatic localized MALT OALs [46]. Because of the indolent behavior of the disease, this strategy can be considered, according to most authors, only when no other treatments are suitable (e.g., frail elderly patients), and this happens rarely.

Intralesional injection of Interferon *α*-2b has been attempted, in conjunctival MALT OALs, obtaining good results [47, 48]. Updated follow up results, however, are not available.

Two pilot studies [49, 50] have reported the successful treatment of orbital MALT OALs by intralesional injection of Rituximab; however, long-term effects are not known yet.

In the last years systemic treatments of primary MALT OALs have gained consideration in literature. Several trials studied the efficacy of antibiotic therapy with doxycycline resulting in response rates around 50–60% [51, 52]. However, the wide variability in prevalence of* Chlamydia psittaci* among different geographical regions, and the lower response rate and durability in respect of other treatments make this therapeutic choice not a standard-of-care, especially in western countries.

The use of systemic chemotherapy, with or without immunotherapy, in primary localized MALT OALs, represents a valid alternative in relapsed patients. First-line chemotherapy could be an option but, since there are not prospective trials encouraging it in localized disease, is not commonly recommended because of the high toxicity profile, especially of the anthracycline-containing regimens. A possible effective and well-tolerated agent is oral chlorambucil, alone [53] or in combination with Rituximab [54], but in the literature the duration of response is not better than other local or less toxic agents.

Only few case series are available on the efficacy of single agent Rituximab immunotherapy in primary localized MALT OALs as showed in Table 2 [7, 8, 11, 12, 55–57]. The results of these studies show that systemic immunotherapy could be of primary importance as first-line treatment, because of the high response rates achieved (comparable to those of local radiotherapy), accompanied also by a favorable tolerability profile. Overall, these studies (all including a small population sample) deeply differ in terms of patients population, line of treatment, staging inclusion criteria, and administration schedule. An issue raised from these data indicates a high rate of relapse with Rituximab monotherapy [1, 8]. In our case series we included only nontreated patients, Ann Arbor staging IE, who underwent 6 cycles of intravenous standard-dose Rituximab every 3 weeks, obtaining good response rates similar to those in literature, and without adverse events except from one case of viral reactivation completely resolved with antiviral therapy. We subsequently treated four of the six patients with intravenous maintenance Rituximab every 2-3 months, with sustained response and without serious toxicity. The whole median follow-up period was 29 months. This treatment strategy was never reported before in primary MALT OALs and may overcome the high rate of relapse showed in literature, especially in the control of local disease, which seems to represent a disadvantage in respect of radiotherapy as first-line management.

In conclusion, we consider Rituximab immunotherapy the therapy of choice in the upfront treatment of primary localized MALT OALs. This induction should be followed by Rituximab maintenance. However, perspective trials in the framework of cooperative groups are needed to establish the exact role of Rituximab and the optimal management of these indolent lymphomas. A direct comparison between radiotherapy and immunotherapy should be performed, to answer the question of the best first-line therapy. Since MALT OALs are associated with a favorable prognosis, therapeutic options are equally effective and preserving patients' quality of life should always be preferred.

## Figures and Tables

**Table 1:** Demographic data, tumor features, and response, in 6 patients with ocular adnexal lymphoma treated with Rituximab immunotherapy.

No.	Age, sex	Eye	Stage	Location	RTX cycles	Interim response	RTX response	RTX maintenance	Survival status	FU months	Final status
1	54/F	OS	IE	C	6	SD	CR	No	Alive	34+	CR
2	62/M	OS	IE	C	6	n.e.	CR	Yes	Alive	27+	CR
3	59/F	OD	IE	C	6	n.e.	CR	Yes	Alive	31+	CR
4	67/F	OS/OD	IE	C	6	n.e.	PR	No	Dead^*∗*^	8	—
5	54/M	OS	IE	O	6	CR	CR	Yes	Alive	31+	CR
6	37/F	OD	IE	O	6	PR	PR	Yes	Alive	9+	PR

M: male; F: female; OS: left eye; OD: right eye; C: conjunctive; O: orbit; SD: stable disease; PR: partial response; CR: complete response; n.e: not evaluated; RTX: rituximab; FU: follow-up.

^*∗*^Lung carcinoma.

**Table 2:** Results by Rituximab in OAL (review of the literature).

	Patients	Diagnosis	Clinical stage	Rituximab dose	Outcome	Longer follows-up (months)
Nückel et al. [36]	2	Relapsed after RT	IE	375 mg/mq once weekly for 4 wks.	1 CR 1 RP	30 and 32

Ferreri et al. [37]	8	5 newly diagnosed 3 relapses	IE (4) IV (4)	375 mg/mq once weekly for 4 wks.	3 CR 2 PR 2 PD 1 SD	Not available

Tuncer et al. [39]	10	Newly diagnosed	IE	375 mg/mq iv every 3 wks. for 6–8 cycles	36% CR 64% PR	31

Zinzani et al. [40]	1	Newly diagnosed	IE	375 mg/mq once weekly for 4 wks.	CR	—

Sullivan et al. [55]	8	Newly diagnosed	—	375 mg/mq once weekly for 4 wks.	5 CR, 2 PR 1 No Res	32

Heinz et al. [56]	1	Newly diagnosed		375 mg/mq once weekly for 4 wks.	CR	—

Mino et al. [57]	10	Newly diagnosed	I-IIE	375 mg/mq every 4 wks. for 6–8 cycles	10 CR	

Present study	6	Newly diagnosed	IE	375 mg/mq every 3 wks. for 6 cycles + maintenance for 2 years	4 CR 2 PR	34
